# How and Why Paediatric Weight Estimation Systems Fail - A Body Composition Study

**DOI:** 10.7759/cureus.7198

**Published:** 2020-03-07

**Authors:** Mike Wells, Lara N Goldstein

**Affiliations:** 1 Emergency Medicine, University of the Witwatersrand, Johannesburg, ZAF

**Keywords:** pawper tape, broselow tape, mercy method, paediatric resuscitation, weight estimation

## Abstract

Background

Weight estimation during medical emergencies in children is essential, but fraught with errors if the wrong techniques are used, which may result in critical drug dosing errors. Individualised weight estimation is required to allow for accurate dosing in underweight and obese children in particular. This study was designed to evaluate the associations between weight estimations from different systems and body composition in order to establish how and why they may perform well or poorly.

Methods

A convenience sample of 332 children aged from one month to 16 years had weight estimations using four age-based formulas: the Broselow™ Pediatric Emergency Tape (Armstrong Medical Industries, Inc., Lincolnshire, IL), the Mercy Method, and the Pediatric Advanced Weight Prediction in the Emergency Room, Extra-large/Extra-long Tape (PAWPER XL) Tape. They also had an assessment of body composition using dual x-ray absorptiometry (DXA). The weight estimates were compared against total body weight (TBW), calculated ideal body weight (IBW), and DXA-measured fat-free mass (FFM). Analyses of associations between age, length, weight estimation outcomes, and body composition were performed.

Results

Age-based formulas were very inaccurate because of the erratic relationship between age and body composition. The Broselow tape estimated IBW well in obese children because of the strong relationship between length and fat-free mass. It predicted TBW poorly in underweight and obese children, however, because of the poor relationship between length and fat mass. The Mercy Method’s performance was unrelated to body composition, but estimated TBW reasonably well and could not predict IBW or FFM. The PAWPER XL Tape’s performance was the most closely associated with body composition and, therefore, achieved an acceptable accuracy for estimations of TBW, IBW, and FFM.

Conclusions

Of the systems evaluated, the PAWPER XL Tape has the best association with body composition and the most accurate estimations of TBW, IBW, and FFM.

## Introduction

The ultimate purpose of the weight estimation systems used during the management of medical emergencies in children is to enable the administration of accurate doses of potentially life-saving drugs [1]. If the weight estimation is inaccurate, or the methodology is prone to error when used during emergency situations, then the child is at risk of medical error [2]. An understanding of how and why these systems can fail would provide insight into which methodologies should be preferred and how they should be used within the context of the overall medical management of the critically ill or injured child. It would also provide understanding into how they could be improved. Malfunction could be as a result of inaccuracy of the estimation technique (producing a high incidence of critical errors), a failure to use the technique correctly (e.g., the Broselow™ Pediatric Emergency Tape used the wrong way round or calculation errors for formulas), a failure to function well in specific subgroups (e.g., underweight and overweight children), or a child falling outside of the weight estimation system’s parameters (e.g., children “too tall for the Broselow Pediatric Emergency Tape”) [3-4].

Some of the most commonly used weight estimation methods include formulas based on age, the Broselow Pediatric Emergency Tape, and newer dual length and habitus-based systems, such as the Mercy Method and the Pediatric Advanced Weight Prediction in the Emergency Room, Extra-large/Extra-long (PAWPER XL) Tape. Some of these methodologies, such as age-based formulas, have already been shown to be inaccurate in previous studies, but the underlying causes have not been well-studied [5-6]. Other techniques, such as the PAWPER XL Tape, have been shown to be accurate in some studies [7-9] but could still potentially be improved, especially as other studies have shown inconsistent accuracy in obese populations [10-11], primarily as a result of inaccurate habitus assessment.

The degree to which weight estimation systems can be considered to be inadequate depends on the standards by which they are judged. With the increasing prevalence of obesity in children, in both high-income as well as low- and middle-income countries, and a high prevalence of underweight children in low- and middle-income countries, weight estimation systems should ideally be able to provide sufficient information to allow correct dosing in children with both normal and extremes of body composition [4]. Although there is still some controversy about the correct dose scalar to use in obese children, the broad consensus is that, while total body weight (TBW) is still used for many drugs, ideal body weight (IBW) is required for the safe dosing of others [12]. This is important because there is some evidence that drug dosing errors might contribute to poorer outcomes in obese children suffering from cardiac arrest [13]. Therefore, while drug dose determination during emergencies may be difficult, a high standard of accuracy is required to ensure patient safety. For underweight children, an accurate estimation of TBW is essential, as IBW may be significantly higher than TBW [4]. Therefore, the ideal weight estimation system needs to be able to predict both TBW and IBW accurately, and an individualised plan should be employed for weight estimation in each child, dependent on their body composition or habitus. 

The primary objectives of this study were to evaluate the vulnerabilities of selected age-based formulas, the Broselow Pediatric Emergency Tape, the Mercy Method, and the PAWPER XL Tape with regards to the prediction of an appropriate weight descriptor for drug dose calculations and to identify how variations in body composition could influence the accuracy of weight estimation. 

## Materials and methods

This was a prospective, cross-sectional study conducted in an academic-aligned hospital in Johannesburg, South Africa, which treats approximately 6000 children per year. Ethics approval for this study was obtained from the Human Research Ethics Committee of the University of the Witwatersrand (approval number M120486).

A convenience sample of 332 children from one month to 16 years of age who presented to the Emergency Department (but did not require emergency treatment) were enrolled between October and December 2015. Exclusion criteria included failure to obtain consent and the inability to obtain critical measurements. 

After basic demographic data were obtained, each child was changed into a hospital gown for the subsequent measurements. Anthropometric measurements of length, mid-arm circumference (MAC) and humerus length were obtained with the child in a supine position (to simulate emergency treatment conditions). Measured weight was obtained using a Tanita SC-240 Body Composition Analyser, following which whole-body dual x-ray absorptiometry (DXA) measurements of body composition were acquired using a Hologic Discovery A Densitometer (software version 12.6). All data were collected by one of the researchers (MW or LG).

TBW was estimated with the Broselow tape [14] and PAWPER XL tape [8] as well as the Mercy Method [15], the Advanced Paediatric Life Support (APLS) formulas, Erker formulas [16], the European Paediatric Life Support (EPLS) formula, and the Best Guess formulas (Table 1). A visual gestalt assessment of habitus was used to classify children for the Erker formulas into “thin,” “normal,” and “thick” categories [16]. Body mass index (BMI), BMI-for-age Z-scores, and an estimate of IBW (using the BMI50 method) were calculated using the Centers for Disease Control (CDC) growth charts [17].

**Table 1:** A Description of the Weight Estimation Methods That Were Included in this Study PAWPER XL: Pediatric Advanced Weight Prediction in the Emergency Room, Extra-large/Extra-long Tape; Wt: weight in kilograms; Z: age in years; z: age in months.

Name	Formula or method	Restrictions
European Paediatric Life Support (EPLS) formula (also known as the old APLS formula)	Wt = (2 × Z) + 8 or Wt = 2 × (Z + 4)	Age restriction 1 to 10 years of age.
Advanced Paediatric Life Support (APLS) formula (also known as the new APLS formula)	Wt = z / 2 + 4	For infants ≤12 months of age.
	Wt = (2 × Z) + 8 or Wt = 2 × (Z + 4)	For children aged 1 to 5 years.
	Wt = (3 × Z) + 7	For children aged 6 to 12 years.
Best Guess formula	Wt = [z + 9) / 2	For infants ≤12 months of age.
	Wt = (2 × Z) + 10 or Wt = 2 × (Z + 5)	For children aged 1 to 5 years.
	Wt = 4 × Z	For children aged 6 to 14 years.
Erker formulas	Wt = (2 × Z) + 6 or Wt = 2 × (Z + 3)	For “thin” children aged 1 to 12 years.
	Wt = (3 × Z) + 6 or Wt = 3 × (Z + 2)	For “normal” children aged 1 to 12 years.
	Wt = (4 × Z) + 6	For “thick” children aged 1 to 12 years.
Broselow tape	An estimate of total body weight can be read directly off the tape laid next to a supine child.	Length restriction 143 cm.
PAWPER XL tape	An estimate of ideal body weight can be read directly off the tape laid next to a supine child. Total body weight can be estimated by adjusting the weight estimation up or down depending on a visual estimation of body habitus. The total body weight estimates can be read off the tape with no need for calculations.	Length restriction 180 cm.
Mercy method	Measurements of humeral length and mid-arm circumference are used to estimate weight. “Partial” weights are read off a table for each measurement and added for an estimation of total body weight.	For children aged 2 to 16 years.

Fat-free mass (FFM), fat-free mass index (FFMI), and fat mass index (FMI) were derived from the DXA data for each child, using proprietary paediatric formulas installed by the manufacturer.

This data was evaluated using an analysis based on a modified Bland-Altman methodology. Each weight-estimation system was compared with TBW, IBW, and DXA-measured FFM using a percentage error analysis. Mean percentage error (MPE), 95% limits of agreement of percentage error, and the percentage of estimations falling within 10% (PW10) and 20% (PW20) of the weight descriptor were the three primary outcome measures used.

Subgroup analyses were performed in three weight categories (< 10 kg, 10 to 25 kg, and >25 kg) and three habitus categories based on BMI-for-age Z-scores (“thin” children Z-score ≤ 2.0, “fat” children Z-score ≥ 2.0, and normal-weight children in between). A subgroup analysis was also performed for those children falling outside the restrictions of the weight estimation systems. A PW10 of > 70% and a PW20 > 95% was considered to be an acceptable accuracy for a weight estimation method [2, 18].

The associations between age, length, and body composition were evaluated using a graphical representation and Pearson correlation analysis, with and without logarithmic transformation, as appropriate.

**Figure 1:**
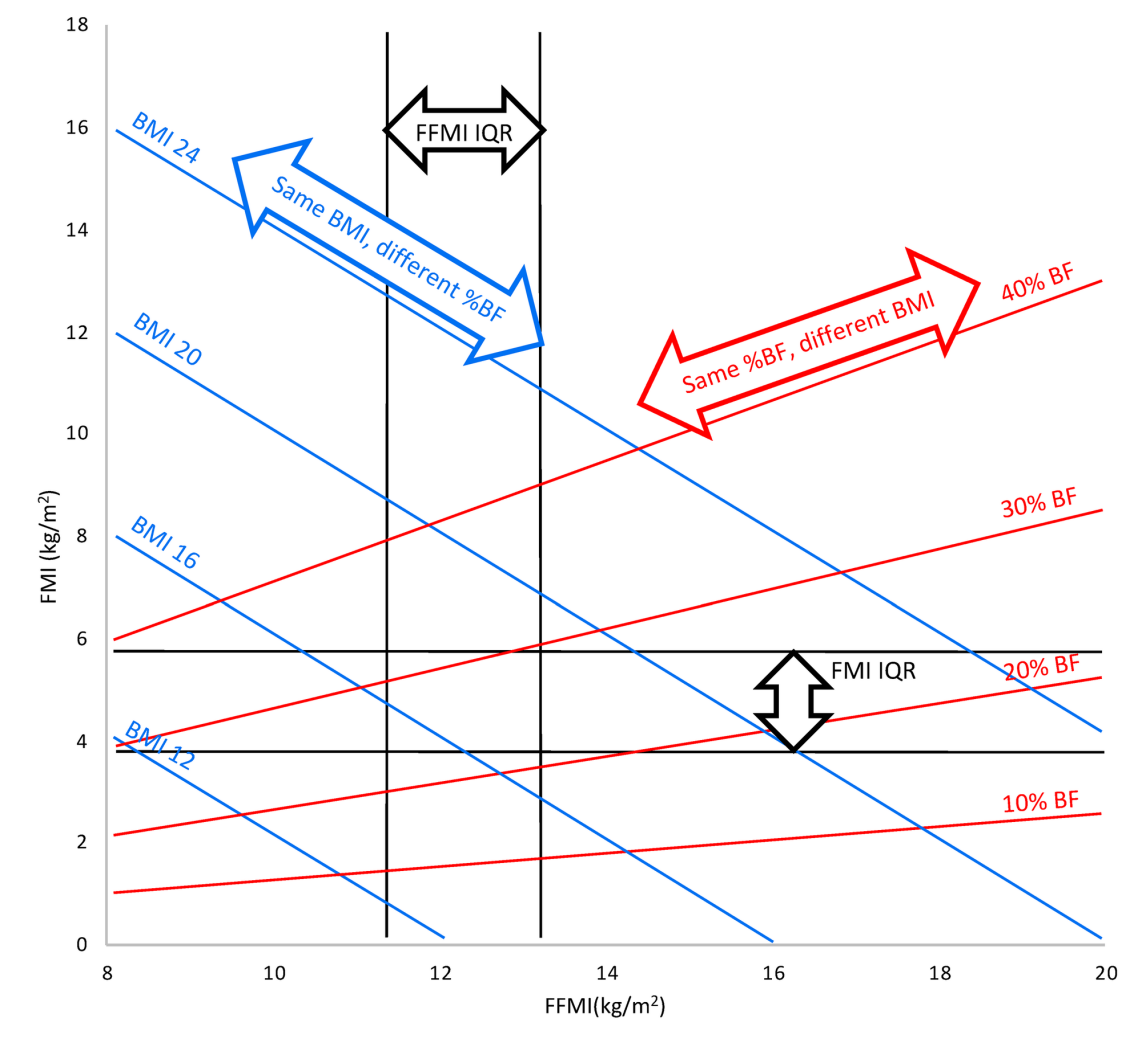
An explanation of the Hattori chart analysis For each child in the study sample, the values of FM (kg) and FFM (kg) measured with DXA were used to calculate two new indices of body composition, normalised for height: FFMI (calculated as FFM/height^2^) and FMI (calculated as FM/height^2^). The values of FFMI and FMI for each child can then be plotted on the chart. The x-axis represents FFMI (kg/m^2^) and the y-axis FMI (kg/m^2^). The contributions of FFMI and FMI to BMI can be easily identified. Isolines of BMI and % body fat are represented by diagonal lines across the chart (in blue and red, respectively). In the absence of adequate reference data for children with respect to FFMI and FMI, the first and third quartiles for FFMI and FMI from the current study population were plotted on the chart. These are represented by the vertical and horizontal black lines, respectively. Four kinds of information can be read off the chart at the same time: FMI, FFMI, BMI, and % body fat, in addition to identifying any individual child’s FFMI and FMI relative to the study population. BF: body fat; BMI: body mass index; DXA: dual x-ray absorptiometry; FFMI: fat-free mass index; FM: fat mass; FMI: fat mass index; IQR: interquartile range

Data from each of the weight estimation systems were plotted on a Hattori chart, according to the percentage error category, to represent the associations between body composition and accuracy of weight prediction [19]. A description of the Hattori chart analysis is shown in Figure 1.

In order to evaluate the accuracy of the assignment of habitus score (HS) for the PAWPER XL, an “ideal” HS was determined for each child (the HS which would result in the best weight estimate at the child’s length), which was compared to the original HS. For comparisons with IBW and FFM, the PAWPER XL HS3 and HS1 weights, respectively, were used.

## Results

Characteristics of study participants

A total of 332 children were included in the study. The basic demographic and body composition data is shown in Table 2. There were a substantial number of underweight children (15.3%) and overweight or obese children (22.3% and 10.2%, respectively). This allowed for the weight estimation systems to be tested over a spectrum of body habitus variations. Subgroup data for thin, normal, and fat children were based on categories derived using the BMI system described by Cole et al. [20]. Since body composition reference data have not been well established in children, and especially in younger children, most analyses in this study made use of pragmatic limits that might affect drug dosing decisions: children were considered to be significantly “fat” when their TBW was > 120% of IBW (which roughly corresponds to a Z-score of +2) as this would require the use of IBW as a drug dosing descriptor for some drugs. Likewise, children were considered significantly “thin” when their IBW was > 120% TBW (which approximates a Z-score of -2) as the use of IBW would result in a critical overdosing error. There was a clinically important number of children whose TBW and IBW differed by more than 20%.

**Table 2:** Description of the Study Population: Demographic Information with Body Composition Data The data is presented for the whole sample as well as for categories of habitus BF: body fat; BMI: body mass index; FFMI: fat-free mass index; FMI: fat mass index; HS: habitus score; IBW: ideal body weight; IQR: interquartile range; TBW: total body weight

		Body mass index category					
	All	Thinness Gr 2	Thinness Gr 1	Normal	Overweight	Obese	Severely obese
Number [n (%)]	332 (100.0)	18 (5.4)	33 (9.9)	173 (52.1)	74 (22.3)	20 (6.0)	14 (4.2)
Sex (male) [n (%)]	154 (46.4)	4 (22.2)	15 (45.5)	93 (53.7)	25 (33.8)	12 (60.0)	5 (35.7)
Age (years) [median (IQR)]	7.2 (4.5, 9.3)	6.4 (3.5, 8.0)	9.1 (5.7, 13.1)	7.3 (4.9, 10.1)	7.8 (5.0, 9.4)	6.7 (4.9, 10.0)	3.0 (1.7, 6.9)
BMI (kg/m^2^) [median (IQR)]	16.7 (15.2, 18.8)	13.2 (12.9, 13.4)	16.3 (15.6, 18.6)	16.2 (15.4, 17.3)	18.6 (17.8, 20.0)	19.9 (19.3, 26.6)	22.6 (20.5, 26.4)
BMI-for-age Z-score [median (IQR)]	0.4 (-0.5, 1.1)	-2.7 (-3.5, -2.2)	0.0 (0.0, 0.0)	0.3 (-0.3, 0.6)	1.4 (1.1, 1.6)	2.1 (2.0, 2.2)	2.9 (2.8, 3.5)
BF (%) [median (IQR)]	27.7 (24.2, 32.0)	23.2 (21.4, 26.7)	26.8 (24.6, 27.7)	25.5 (23.4, 28.6)	32.3 (29.7, 33.7)	34.8 (31.3, 37.8)	38.3 (34.9, 45.2)
FMI (kg/m^2^) [median (IQR)]	4.6 (3.7, 5.7)	3.0 (2.7, 3.4)	4.5 (4.3, 4.7)	4.2 (3.7, 4.9)	5.9 (5.5, 6.7)	6.8 (6.1, 10.3)	8.8 (7.1, 11.2)
FFMI (kg/m^2^) [median (IQR)]	12.2 (11.3, 13.2)	10.0 (9.6, 10.3)	11.8 (11.5, 14.1)	12.1 (11.5, 12.7)	12.8 (12.3, 14.1)	14.4 (12.6, 15.1)	13.9 (13.4, 15.2)
IBW (kg) [median (IQR)]	23.2 (17.7, 30.7)	22.2 (14.9, 23.5)	26.4 (21.2, 43.0)	22.8 (18.3, 31.5)	25.9 (18.9, 31.5)	23.7 (18.2, 35.9)	14.2 (11.3, 35.9)
IBW > 120% TBW [n (%)]	46 (13.9)	18 (100)	28 (84.8)	-	-	-	-
TBW > 120% IBW [n (%)]	58 (17.5)	-	-	1 (0.6)	25 (33.8)	18 (90.0)	14 (100)
Habitus score [median (IQR)]	3 (3, 4)	2 (2, 2)	3 (3, 3.75)	3 (3, 4)	4 (4, 4.8)	4.5 (4, 5)	5 (5, 6)
TBW (kg) [median (IQR)]	23.4 (17.6, 33.8)	18.3 (12.3, 20.0)	26.6 (21.1, 43.1)	23.4 (18.4, 31.4)	30.6 (21.6, 38.8)	30.8 (21.3, 56.3)	19.0 (15.5, 39.3)

Main results

The performances of each of the weight-estimation systems against TBW, IBW, and DXA-measured FFM are shown in Table 3. With regards to estimating TBW, the overall accuracy of the regular age-based formulas was very poor (PW10 ranging from 29.7 to 43.1%) with even the habitus-modified Erker formula achieving a PW10 of only 50.0%. The Broselow tape and the Mercy method achieved an intermediate degree of accuracy (PW10s of 52.1 and 63.9%, respectively) and the PAWPER XL tape achieved a high degree of accuracy (PW10 83.4%). The age-formulas and the Mercy method did not estimate IBW accurately in obese children (PW10_IBW_ 24.1% to 39.7% and 12.1%, respectively). The Broselow tape and the PAWPER XL tape (using the HS3 weight to predict IBW) both predicted IBW well (PW10_IBW_ 72.4 and 87.9%, respectively), with the PAWPER XL tape accurately identifying all the obese children (those scored as an HS ≥ 5). None of the other systems had a mechanism to identify obese children. When it came to predictions of FFM, only the PAWPER XL tape (using the HS1 weight to predict FFM) and the EPLS formula achieved a PW10_LBW_ of greater than 50%, with PW10s of 82.4% and 58.6%, respectively.

**Table 3:** Accuracy Outcomes of the Weight Estimation Systems Evaluated in This Study The data is presented for the whole sample as well as by subgroups of weight, habitus, and two special categories (length > 145 cm and HS > 5). For the purposes of this evaluation, children were defined as “thin” if TBW was less than 90% of IBW, “fat” if TBW was greater than 120% of IBW, and “normal” for the remainder. The accuracy of the weight estimation systems was specifically evaluated in children with length > 145 cm as those children comprise the subgroup of children too tall for the Broselow tape. The subgroup of children with HS > 5 was important because this represents severely obese children. The PAWPER XL tape has a defined mechanism for predicting IBW and FFM (or lean body weight (LBW)). The HS3 weight is used to predict IBW and the HS1 weight is used to predict FFM. Since these dosing scalars are only intended to be used in obese children, the data were only calculated for the obese children in the sample. BMI: body mass index; FFM: fat-free mass; HS: habitus score; IBW: ideal body weight; LBW: lean body weight; LLOA: Bland-Altman lower limit of agreement; MPE: mean percentage error; PAWPER XL: Pediatric Advanced Weight Prediction in the Emergency Room, Extra-large/Extra-long; PW10: percentage of weight estimates within 10% of actual weight; PW20: percentage of weight estimates within 20% of actual weight; TBW: total body weight; ULOA: Bland-Altman upper limit of agreement

			Total body weight (TBW)					Ideal body weight (BMI_50_)					Measured FFM (LBW)				
		N	PW10	PW20	MPE	LLOA	ULOA	PW10	PW20	MPE	LLOA	ULOA	PW10	PW20	MPE	LLOA	ULOA
Broselow tape	All	332	52.1	80.1	-5.1	-36.9	26.6	80.7	94.0	-0.7	-20.6	19.3	6.6	22.0	31.9	-4.9	68.7
	< 10 kg	11	72.7	81.8	6.7	-18.1	31.4	100	100	3.1	-9.7	16.0	0	2.0	43.7	18.2	69.1
	10-25 kg	168	62.5	90.5	0.9	-21.9	23.7	92.4	100	1.3	-9.6	12.2	2.8	13.0	36.2	9.7	62.8
	> 25 kg	153	39.2	68.6	-12.6	-46.8	21.5	63.8	85.5	-3.5	-30.7	23.6	24.2	66.7	8.7	-27.9	45.4
	Thin	46	26.1	63.0	15.3	-2.9	33.5	87.0	95.7	-1.5	-16.0	13.0	0	6.5	52.2	24.1	80.2
	Normal	218	68.9	92.5	-3.9	-25.4	17.5	81.6	95.2	-0.6	-19.6	18.4	5.3	17.1	32.1	2.4	61.8
	Fat	58	6.9	44.8	-26.0	-53.1	1.0	72.4	87.9	-0.2	-26.7	26.2	17.2	53.4	14.9	-21.7	51.6
	> 145 cm	48	16.7	41.7	-25.9	-59.5	7.7	31.3	58.3	-17.9	-43.0	7.1	31.3	60.4	17.5	-30.6	65.5
	HS > 5	15	0	0	-36.4	-56.4	-16.4	73.3	80.0	-3.5	-26.2	19.3	46.7	93.3	8.2	-17.8	34.3
Mercy method	All	332	63.9	94.3	-6.7	-23.1	9.6	51.5	79.8	0	-39.0	39.1	6.3	27.1	31.0	-3.9	65.9
	< 10 kg	11	54.5	90.9	-4.2	-27.4	19.0	70.0	70.0	-1.3	-32.6	30.0	14.0	38.0	28.6	-8.1	65.2
	10-25 kg	168	64.9	92.3	-7.5	-23.8	8.9	51.1	82.1	-4.3	-35.2	26.7	6.0	26.9	29.6	-0.6	59.7
	> 25 kg	153	63.4	96.7	-6.1	-21.8	9.7	50.7	77.5	5.8	-40.2	51.8	1.5	19.7	37.5	-7.0	82.0
	Thin	46	78.3	93.5	-3.7	-19.9	12.5	15.2	65.2	-17.7	-30.8	-4.6	4.3	28.3	27.2	1.7	52.6
	Normal	218	62.3	94.7	-8.0	-22.6	6.6	68.9	93.9	-4.4	-23.5	14.6	7.0	31.6	26.8	1.4	52.1
	Fat	58	58.6	93.1	-4.2	-24.6	16.2	12.1	36.2	31.6	-14.2	77.4	5.2	8.6	50.6	3.7	97.4
	> 145 cm	48	70.8	100	-4.8	-18.4	8.7	52.1	68.8	9.4	-40.3	59.1	8.3	16.7	59.9	-51.1	171
	HS > 5	15	53.3	93.3	-1.1	-25.2	22.9	0	6.7	51.5	1.7	101	0	0	69.1	27.3	111
PAWPER XL tape TBW	All	332	83.4	98.5	1.1	-13.9	16.1	62.7	89.8	6.1	-15.7	27.9	0	5.1	29.2	17.0	41.4
	< 10 kg	11	54.5	100	8.7	-7.1	24.4	50.0	100	8.8	-7.0	24.6	0	0	33.8	23.6	44.0
	10-25 kg	168	86.3	100	2.0	-10.4	14.3	71.7	95.1	3.8	-13.8	21.4	0	5.6	28.2	17.9	38.5
	> 25 kg	153	82.4	96.7	-0.4	-17.1	16.3	51.4	81.9	9.1	-16.5	34.7	0	7.6	29.0	13.1	44.8
	Thin	46	67.4	97.8	7.8	-3.2	18.9	65.2	97.8	-7.9	-25.1	9.2	0	2.2	29.9	17.5	42.4
	Normal	218	91.7	100	1.5	-10.0	13.1	77.2	99.1	5.0	-7.5	17.4	0	5.7	28.3	17.3	39.3
	Fat	58	63.8	93.1	-6.0	-24.5	12.5	3.4	46.6	21.9	4.4	39.4	0	5.2	31.9	17.3	46.6
	> 145 cm	48	83.3	97.9	-0.3	-16.8	16.2	52.1	70.8	9.6	-24.6	43.8	0	16.7	34.9	0.3	69.6
	HS > 5	15	53.3	86.7	-6.2	-30.0	17.7	0	13.3	30.4	16.2	44.7	0	0	38.0	27.5	48.6
PAWPER XL tape IBW	Obese	34						87.9	100	4.8	-4.5	14.1					
PAWPER XL tape LBW	Obese	34											94.1	100	0.1	-11.9	12.2
APLS formula (new Advanced Paediatric Life Support formula)	All	332	43.1	68.1	2.0	-38.0	41.9	44.1	85.3	8.1	-15.0	31.3	2.9	19.2	30.3	8.4	52.3
	< 10 kg	11	27.3	63.6	13.8	-13.4	41.1	20.0	100	13.0	-6.5	32.4	0	6.0	32.5	15.2	49.9
	10-25 kg	168	45.8	64.3	3.7	-34.3	41.7	49.5	81.5	5.6	-20.2	31.5	2.8	20.8	31.4	8.6	54.2
	> 25 kg	153	35.9	64.1	-1.2	-40.6	38.1	32.6	77.5	11.6	-5.4	28.6	4.5	18.2	23.0	-1.8	47.7
	Thin	46	21.7	34.8	22.2	-2.0	46.4	28.3	78.3	9.4	-16.0	34.8	0	4.3	40.9	18.8	63.1
	Normal	218	49.1	76.8	4.6	-21.3	30.4	45.2	78.9	8.1	-15.2	31.4	0.9	12.7	30.7	8.1	53.4
	Fat	58	22.4	37.9	-26.5	-68.1	15.1	37.9	87.9	7.3	-10.9	25.5	12.1	50.0	19.4	-1.0	39.9
	> 145 cm	38	44.8	75.9	-10.9	-60.3	38.4	79.3	96.6	3.1	-11.1	17.4	0	3.4	31.6	14.1	49.2
	HS > 5	15	23.1	23.1	-47.0	-102	7.6	23.1	92.3	5.2	-20.4	30.9	38.5	69.2	14.5	-10.9	40.0
EPLS formula (European Paediatric Life Support formula)	All	297	40.4	60.5	-17.5	-75.9	41.0	53.0	79.5	-9.0	-39.6	21.5	22.9	50.3	16.6	-16.8	50.1
	< 10 kg	5	0	54.5	21.1	-1.3	43.5	0	70.0	20.6	2.2	39.1	0	6.0	33.6	14.2	53.0
	10-25kg	168	63.1	86.9	-2.6	-30.3	25.1	75.5	98.4	-2.1	-18.3	14.1	16.7	49.5	19.9	-0.2	39.9
	> 25 kg	124	18.3	32.0	-36.6	-98.2	25.0	26.8	55.1	-20.4	-50.2	9.4	60.6	86.4	-6.8	-37.8	24.2
	Thin	41	50.0	80.4	8.0	-17.7	33.7	58.7	84.8	-7.4	-34.0	19.2	6.5	15.2	30.0	8.1	51.9
	Normal	205	48.2	71.1	-12.1	-48.6	24.5	55.3	79.4	-7.8	-37.8	22.2	17.1	50.0	18.5	-8.2	45.1
	Fat	52	1.7	3.4	-58.8	-129	10.8	39.7	75.9	-15.2	-48.3	17.8	58.6	79.3	-1.2	-39.0	36.6
	> 145 cm	20	2.1	12.5	-56.0	-127	15.3	0	6.3	-35.9	-61.8	-10.0	54.2	81.3	3.7	-23.6	31.0
	HS > 5	13	0	0	-87.2	-157	-17.0	20.0	60.0	-21.4	-49.4	6.6	53.3	80.0	-8.6	-38.6	21.3
Erker formula	All	297	50.0	82.6	2.8	-26.4	32.0	37.6	76.2	8.1	-21.0	37.2	3.0	15.1	30.6	11.8	49.5
	< 10 kg	5	45.5	54.5	7.4	-1.2	15.9	0	50.0	12.3	-0.4	25.0	2.0	2.0	31.0	8.1	53.8
	10-25 kg	168	52.4	83.3	4.6	-23.3	32.5	41.8	79.3	6.7	-22.2	35.5	3.7	15.7	31.2	11.0	51.4
	> 25 kg	124	36.6	65.4	0.2	-27.6	28.0	25.4	55.1	10.3	-17.4	38.0	0	15.2	27.2	-2.3	56.7
	Thin	41	67.4	84.8	1.8	-19.1	22.7	30.4	69.6	-14.1	-38.3	10.1	6.5	26.1	25.0	1.8	48.3
	Normal	205	43.0	75.9	6.4	-17.2	30.0	36.4	75.0	9.9	-11.6	31.3	2.2	8.8	32.1	6.9	57.3
	Fat	52	34.5	58.6	-10.5	-44.8	23.8	25.9	41.4	18.6	-7.1	44.3	1.7	22.4	29.3	2.5	56.1
	> 145 cm	20	60.0	85.0	-9.6	-42.6	23.5	60.0	60.0	6.6	-29.3	42.5	5.0	20.0	35.3	-0.5	71.1
	HS > 5	13	7.7	38.5	-20.6	-67.3	26.1	23.1	23.1	22.1	-3.4	47.7	0	30.8	29.8	6.1	53.6
Best Guess formula	All	332	34.5	65.5	9.2	-26.6	45.0	26.1	69.3	15.0	-3.2	33.2	0.9	6.2	35.4	15.9	54.9
	< 10 kg	10	18.2	27.3	23.4	-3.6	50.4	20.0	30.0	22.3	-1.1	45.8	0.0	0.0	41.7	27.3	56.0
	10-25 kg	169	31.5	66.7	12.6	-16.4	41.6	31.5	74.5	13.4	-5.2	32.0	0.5	7.9	35.5	16.1	55.0
	> 25 kg	153	36.6	62.7	4.1	-35.1	43.3	17.4	60.1	16.7	-1.0	34.4	3.0	4.5	29.4	3.7	55.1
	Thin	46	4.3	26.1	27.8	9.3	46.4	23.9	58.7	15.7	-3.4	34.9	0.0	0.0	45.2	29.8	60.6
	Normal	218	37.7	71.5	11.9	-10.5	34.4	27.2	66.2	15.1	-4.2	34.5	0.0	1.8	35.9	16.5	55.4
	Fat	58	39.7	62.1	-18.0	-52.6	16.7	19.0	77.6	13.7	-0.9	28.4	5.2	27.6	25.0	4.7	45.2
	> 145 cm	48	34.2	73.7	-0.3	-43.5	42.9	42.1	97.4	11.1	-2.2	24.4	2.6	5.3	30.1	14.1	46.2
	HS > 5	15	26.7	26.7	-34.2	-82.1	13.7	33.3	80.0	12.9	-5.0	30.9	13.3	40.0	22.0	0.0	43.9

The PAWPER XL tape, the Mercy method, and the APLS formula were able to provide valid weight estimations for all children in the study sample, with the Broselow tape, the EPLS, Erker, and Luscombe formulas outside of their restrictions in 14.5%, 10.5%, 10.5%, and 22.9%, respectively. For the subgroup of children “too tall” for the Broselow tape (those with a length > 145 cm), only the Mercy method and the PAWPER XL tape maintained acceptable accuracy.

**Figure 2:**
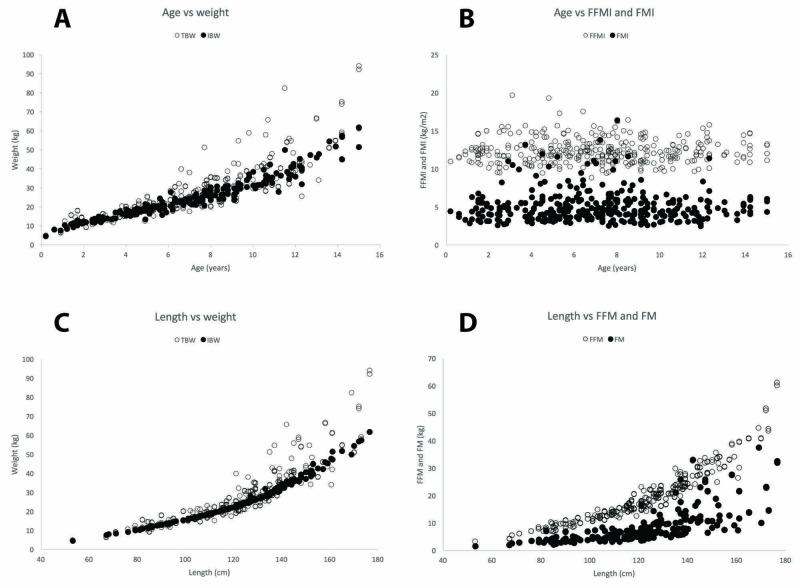
The relationships between age, length, body weight, and body composition found in the study sample Panel A: the relationship between age and total and ideal body weight. The open circles represent total body weight and the filled circles represent ideal body weight; Panel B:  the relationship between age and fat-free mass index and fat mass index. The open circles represent the fat-free mass index and the filled circles represent the fat mass index; Panel C: the relationship between length and total and ideal body weight with length. The open circles represent total body weight and the filled circles represent ideal body weight; Panel D: the relationship between length and fat-free mass and fat mass. The open circles represent fat-free mass and the filled circles represent fat mass. FFM: fat-free mass; FFMI: fat-free mass index; FM: fat mass; FMI: fat mass index; IBW: ideal body weight; TBW: total body weight

The relationships between age, length, and body composition components (including FFM and FM components) are shown in Figure 2 (Panels A-D). The relationship between age and weight (Panel A) was weaker with TBW than with IBW (r^2 ^= 0.74 and r^2^ = 0.91 for TBW and IBW, respectively, p < 0.001). This difference was greatest in older children. Once the effect of height was removed from the age (Panel B) by expressing FFM and FM as indices (FFM/height^2^ and FM/height^2^), the very poor association between age and FFMI and FMI was exposed (r^2 ^= 0.26 and r^2^ = 0.03, respectively, p < 0.05 and p = 0.8, respectively). Length and weight were strongly correlated (Panel C), although the relationship between TBW and length was weaker than between IBW and length (r^2 ^= 0.77 and 0.93 for TBW and IBW, respectively, p < 0.001). This association was reflected in the association between length and FFM and FM (Panel D). Length was strongly correlated with FFM (r^2 ^= 0.86, p < 0.001) but far less well with FM (r^2 ^= 0.48, p < 0.001). This relationship held true when evaluating length against logarithmic transformations of FFM and FM (r^2 ^= 0.96 and r^2^ = 0.66, respectively, p < 0.001). In summary, the relationship between age and TBW and IBW was much weaker than that between length and weight descriptors. While the length and IBW and the length and FFM were strongly correlated, the relationship with TBW and FM was significantly weaker.

**Figure 3:**
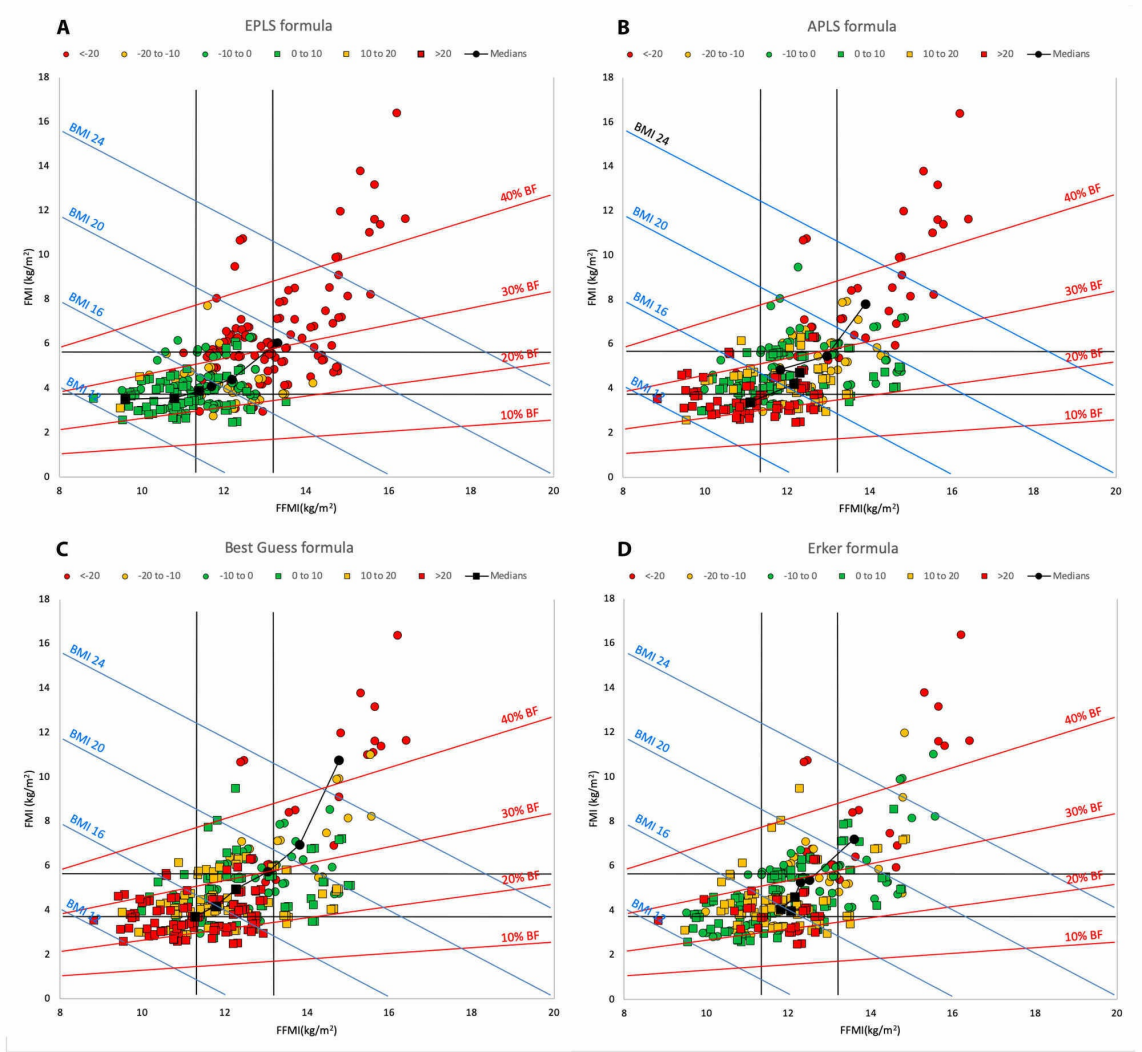
Hattori charts of the study population showing outcomes of total body weight estimation by the age-based formulas Panel A: outcomes of total body weight estimation by the European Paediatric Life Support (EPLS) age-based formula; Panel B: outcomes of total body weight estimation by the new Advanced Paediatric Life Support formula (APLS) age-based formula; Panel C: outcomes of total body weight estimation by the Best Guess age-based formula; Panel D: outcomes of total body weight estimation by the Erker age-based formula. Square markers represent an overestimation of weight and round markers represent an underestimation of weight. Markers with a green fill indicate a weight estimation accuracy of within 10% of actual weight; orange markers an accuracy of between 10% and 20% of actual weight; and red markers an error of greater than 20%. The medians for each error category are shown in black. BF: body fat; BMI: body mass index; FFMI: fat-free mass index; FMI: fat mass index

Figure 3 (Panels A to D) illustrates the associations between body composition and the accuracy of the age-based weight estimation systems in the Hattori chart format. The EPLS formula (Panel A) was the most accurate in children with a low BMI, with no discernible difference in discriminating between FMI or FFMI. In contrast to this, the APLS formula (Panel B) and the Best Guess formula (Panel C) showed a substantially greater overestimation of weight but could not differentiate between children with low and high BMI. The Erker method (Panel D) had a far greater accuracy at all values of BMI than the other formulas. It also, however, produced some large overestimations of weight, even in children of normal weight. In summary, the age-based formulas were all inaccurate but with different biases. The EPLS formula underestimated weight in higher-BMI children, while the APLS and Best Guess formulas overestimated the weight of low-BMI children. The Erker formula had less relationship with body composition but still failed to predict weight accurately.

**Figure 4:**
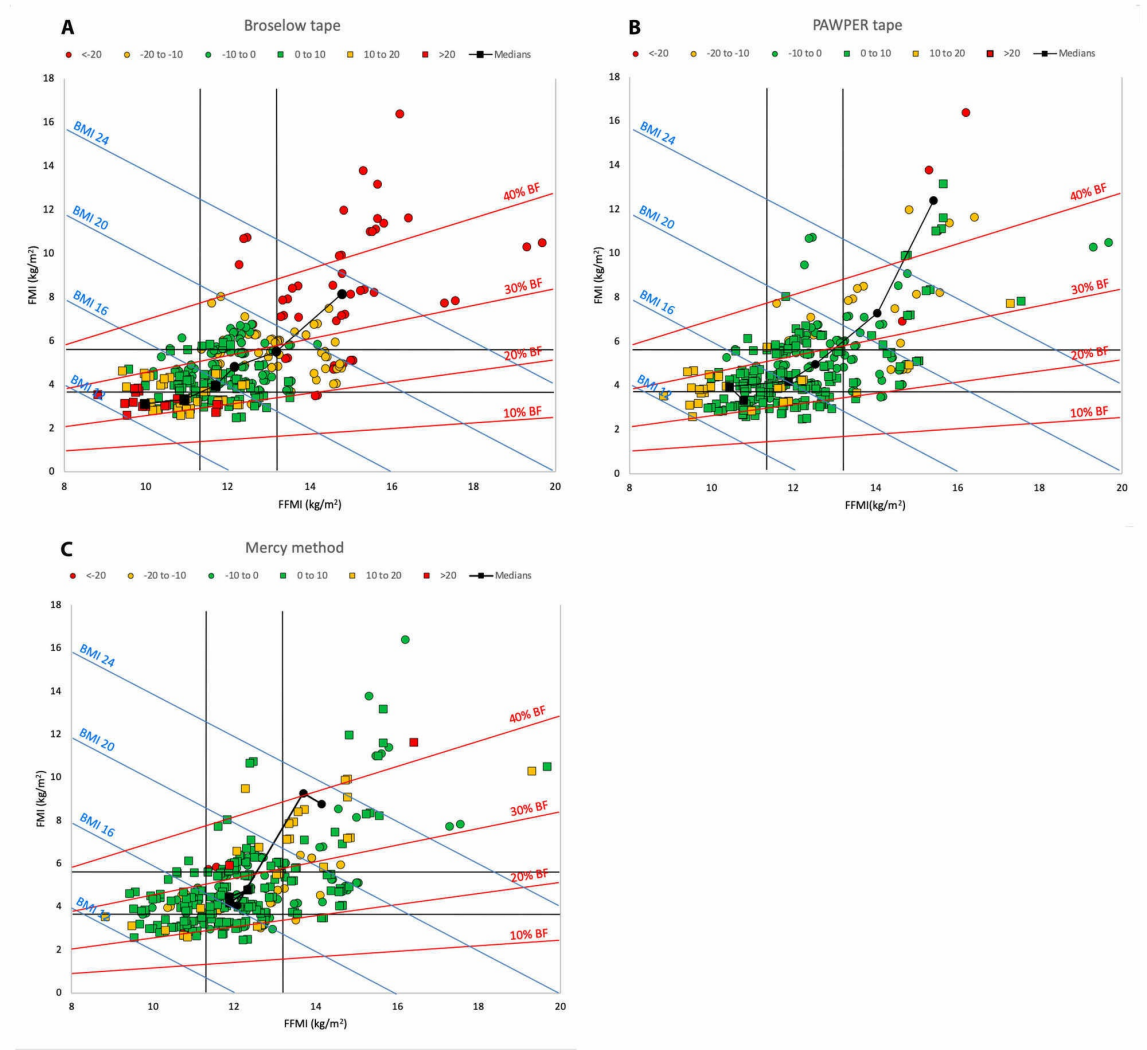
A Hattori chart of the study population showing outcomes of total body weight estimation by the length and length- and habitus-based methods Panel A: outcomes of total body weight estimation by the Broselow tape; Panel B: outcomes of total body weight estimation by the PAWPER XL tape; Panel C: outcomes of total body weight estimation by the Mercy method Square markers represent an overestimation of weight and round markers represent an underestimation of weight. Markers with a green fill indicate a weight estimation accuracy of within 10% of actual weight; orange markers an accuracy of between 10% and 20% of actual weight; and red markers an error of greater than 20%. The medians for each error category are shown in black. BF: body fat; BMI: body mass index; FFMI: fat-free mass index; FMI: fat mass index; PAWPER XL tape: Paediatric Advanced Weight Prediction in the Emergency Room extra large/extra long tape

Figure 4 (Panels A to C) shows the Hattori charts of the length and dual length- and habitus-based methods. The Broselow tape’s performance (Panel A) showed good accuracy in children in the central ranges of FFMI and FMI, but poor results at the extremes of habitus. Both the PAWPER XL tape (Panel B) and the Mercy method (Panel C) revealed a substantially better performance than the other methods at all ranges of FFMI and FMI. While the PAWPER XL tape still showed some inaccuracies at the far extremes, especially of FMI, the Mercy method’s performance was virtually independent of habitus, even at the extremes. In summary, these methods showed fewer critical errors in weight estimation than age-formulas. The Broselow tape showed errors in all children with higher or lower than average BMI, while the PAWPER XL tape showed the same errors only at the extremes of habitus. The performance of the Mercy method showed almost no relationship to body habitus or composition.

**Figure 5:**
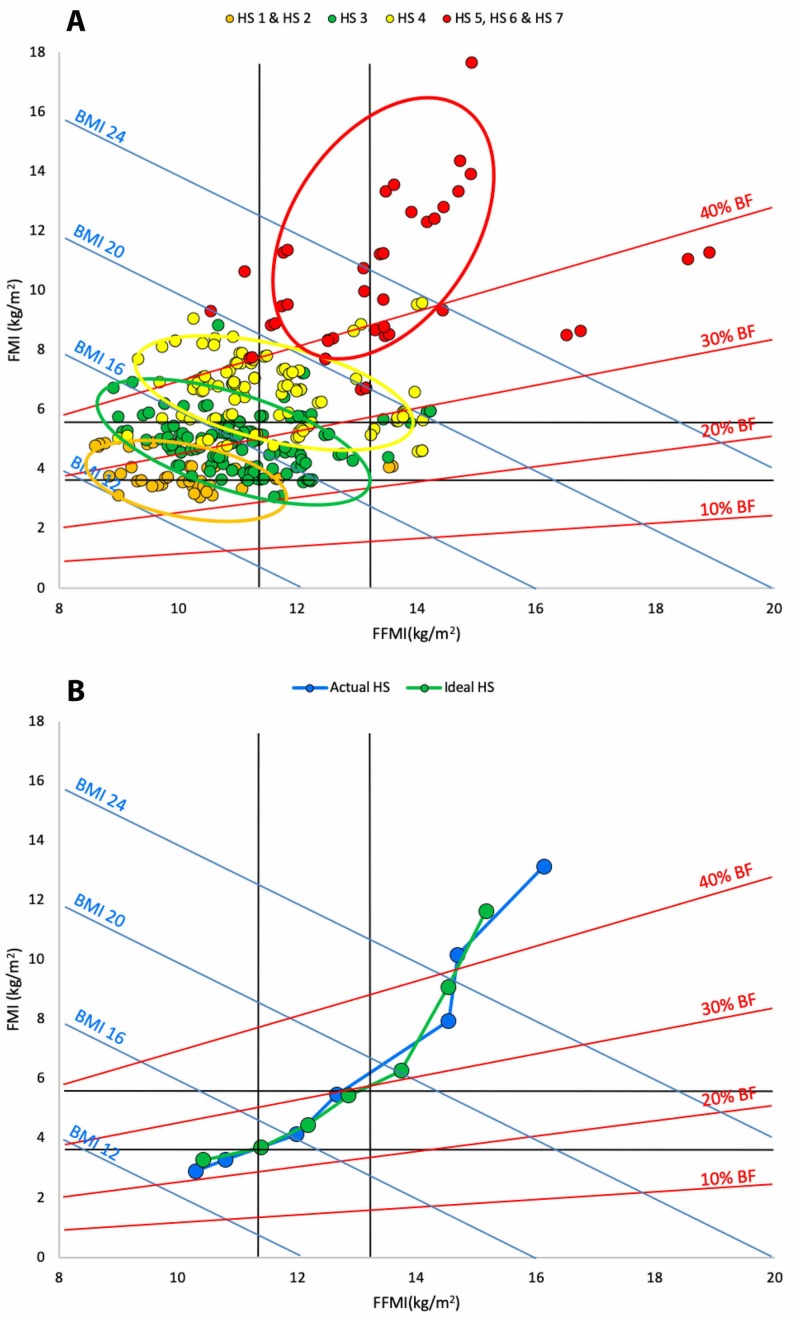
A Hattori chart of the study population showing outcomes of habitus score assignment for the PAWPER XL tape Panel A: the actual habitus score assignments. Orange markers represent HS1 and HS2; green markers represent HS3; yellow markers represent HS4 and red markers represent HS5 and above. The coloured ellipses approximate the 95% limits of agreement for the distribution of each habitus score group; Panel B: a Hattori chart of the FFMI and FMI medians of assigned habitus scores for the PAWPER XL tape. The chart shows the median FFMI and FMI of the actual (blue) and ideal (green) habitus scores (from HS1 to HS7, left to right). BF: body fat; BMI: body mass index; FFMI: fat-free mass index; FMI: fat mass index; HS: habitus score; PAWPER XL tape: Paediatric Advanced Weight Prediction in the Emergency Room extra large/extra long tape

Figure 5 (Panels A and B) contains Hattori charts showing the distribution of habitus scores assigned to the study population. The data showed a good correlation between FMI and HS (r^2 ^= 0.70) and a lesser correlation with FFMI (r^2 ^= 0.43). It also shows a comparison between the actual HS assigned in this study and the “ideal” post hoc HS. In this analysis 51.2% of scores remained unchanged, 41.6% differed by one HS point and the remaining 7.2% differed by two points. In summary, habitus scores accurately represented differences in body composition, especially changes in FM. Ideal and actual habitus score assignments were similar except at extremes of obesity.

## Discussion

Age and length as predictors of TBW, IBW, FFM, and FM

The relationship between age and weight and length and weight is fundamental to the ability of these variables to predict weight. Length was clearly more closely associated than age with TBW, as has been shown previously [21], as well as with IBW and FFM. Once length was removed as a confounding variable, the effect size of the correlation between age and weight was seen to be small. In terms of length, the association with FFM was much closer than with FM, which further explains why one dimensional length-based systems (such as the Broselow tape) were able to predict IBW well but did not predict TBW well because of the potential large variations in FM at any given length [22]. Although length was demonstrably superior to age as a weight-estimation variable, some method of assessing habitus is clearly also required to account for differences in FM between children.

**A note of caution:** although FFM, lean body weight (LBW), and IBW are frequently used interchangeably as an appropriate scalar for drug dosing in obese children, they are not identical. IBW has no true biological validity but has been shown to be similar to FFM in older children [23]. The relationship between LBW and FFM is also unclear in children, but LBW is probably 5% to 10% higher than FFM and is the true “pharmacological scalar” that is desired for calculating doses for hydrophilic medications in obese children [24]. How this should best be translated into clinical practice is, as yet, undetermined.

Interpreting the Hattori chart analysis of weight estimation systems

For the unmodified age-based formulas, the EPLS formula was reasonably accurate only in children with an FFMI and FMI at the lower end ranges; the opposite was true of the Best Guess formula, with the APLS formula falling somewhat between the two. Differences between the age formulas were, essentially, whether the major errors were greater in lower BMI children (APLS and Best Guess formulas) or in higher BMI children (EPLS formula). The degree of weight estimation error for the formulas was only poorly associated with body composition, supporting the findings that age is much less predictive of body weight than length. The habitus-modified Erker formula, although more accurate than the other formulas, showed the most random association between body composition and accuracy. This strongly suggests that, despite a good theoretical basis for this system, it will not be able to achieve adequate accuracy.

The Broselow tape: the most accurate weight estimations were in the central zone (the “normal weight” child), with critical inaccuracies at increasing FFMI and FMI and decreasing FFMI. This was typical of what might be expected - a good relationship between length and weight but variations in FFM/FM not accounted for [22]. Most of the inaccurate estimations were underestimations (from higher than average FMI, and to a lesser extent, FFMI). When considering what the body composition analysis revealed in terms of how the various methods failed, it was clear that the age formulas and Broselow tape were unable to produce accurate weight estimations outside of their narrow area of calibration. Unless these methods are restricted to patient populations that fall within these narrow limitations, they should not be used if better methods are available.

The Mercy method: this method’s performance showed almost no association with body composition. Almost all error categories had FFMI/FMI medians within the central region, with the exceptions of the few children with > 10% weight overestimation who were in the high FFMI/FMI sector. This pattern indicated that some other factor, unrelated to habitus, was the root cause of weight estimation error, such as measurement errors, regional aberrations in body composition, or unknown factors. While the Mercy method was able to accurately predict weight in children of all different body compositions, its failures bore no relation to extremes of body composition. This was, therefore, not a “calibration” error, which would make it difficult to identify specific vulnerabilities and devise methods to improve accuracy.

The PAWPER XL tape: this method showed poor accuracy only at extremes of FFMI and FMI. This was in contrast to the Broselow tape, which showed poor estimation at smaller deviations from the medians. This indicated that the PAWPER XL tape was better “calibrated” as it accounted for a greater degree of variation in body composition. The pattern of inaccuracies further indicated that the tape could potentially be improved with better weight estimation at extremes of body habitus: “fatness” (high FMI) was underestimated in the higher habitus scores, but “slimness” (low FFMI) was under-recognised in the lower habitus scores.

Putting it all together

Age-based Formulas Failed Badly Because of Large Variability of Weight-for-age (FFM and FM)

In this study, the strength of the association between age and TBW or IBW was intermediate at best. This was because the association of age with body composition (FFMI and FMI) was very weak, which was evident once length was removed as a confounding variable. This strongly suggests that it is unlikely that age can ever be used to accurately estimate weight and will always be inferior to length-based systems [25]. The association of age with both FFMI and FMI was weak enough to suggest that neither TBW nor IBW would be able to be accurately predicted. Although the formulas were most accurate in the 10 to 25 kg category (as has previously been found), this accuracy never achieved acceptable levels (PW10 > 70% and PW20 > 95%) as the variability of FFMI and FMI remained consistent across the age range of the population [26]. No age formula has ever been shown to perform satisfactorily well in any previous study (best performances: PW10 45% to 55%) [2]. The new Erker formula, which is habitus-modified, failed to deliver on the potential showed in its theoretical development [16]. This error was predominantly one of modest overestimation of weight in children with normal habitus and modest underestimation of weight in fat children. There were, however, relatively fewer critical errors (estimation error > 20%) than with the other formulas. The biggest weakness with this system was that, at any age, children could have the same habitus but very different lengths (and, therefore, weights) that cannot be accounted for by this system. It is unlikely that further calibration will improve this system significantly.

The Broselow Tape Fails Because of Large Variability of FM for Length

Numerous studies from across the world have confirmed that the tape overestimates weight in underweight populations (often to a dangerous degree) and underestimates weight in populations with a high prevalence of obesity [2, 26]. Although the relationship between length and weight was far stronger than that between age and weight, the relationship between length and FM was far more inconsistent and increasingly variable with increasing length. This variability was sufficient to account for the relatively poor performance of the tape. It also explains why the tape predicted TBW well in children of “normal” habitus, but poorly in all others. This is why researchers have successfully proven the possibility of using alternative methods of habitus assessment to improve the accuracy of the Broselow tape [27, 28].

The present study found that the relationship between length and FFM was strong, which explained the excellent association between length and IBW for the Broselow tape. Importantly, however, this is only relevant for obese children (with respect to drug dosing for hydrophilic drugs), but the Broselow tape as yet has no validated mechanism for identifying obese children. This needs to be considered if the Broselow tape is used in overweight or obese children.

The Mercy Method Fails Because of Non-habitus-related Factors

Generally, the Mercy method predicted TBW well across the age and habitus spectrum but was weakest in infants and obese children. Critical errors were very uncommon. It predicted IBW poorly, especially in obese children in whom IBW would be required, but this was not surprising, as the method was not designed to predict IBW. The system also had no mechanism to identify obese children for whom an alternate weight-descriptor (such as IBW) might be required. The inaccurate estimations incurred by this method were not specifically attributable to habitus, although the poorest accuracy was in obese children (with a mixture of under- and overestimation of weight, however). Part of the explanation might be that, in this study, children were measured in the supine position (simulating how it would be used in medical emergencies), unlike previous studies, which might have led to less accurate measurements [29]. Other studies, not by the developers of the system, have also shown a substantially poorer accuracy than in the original studies [2, 7, 9]. Nonetheless, the Mercy method remains one of the most accurate systems available today, although not ideally suited for use in the Emergency Department.

The PAWPER XL Tape Fails Because of Incorrect Assignment of Habitus Scores

Generally, the PAWPER XL tape predicted TBW, IBW, and FFM extremely well but was weakest in infants (overestimating weight) and morbidly obese children (underestimating weight). The rate of critical estimation errors for all weight descriptors was the lowest amongst all the weight estimation systems. The errors of estimation appeared to be directly related to body habitus, which suggested that an improvement in the assessment of habitus might further improve the tape’s “calibration” and, therefore, accuracy. Studies in obese populations in the United States have also demonstrated this vulnerability to inaccurate HS assignment [11]. The “ideal” HS assignments showed that the habitus estimations were frequently more moderate than the ideal scores and that it was theoretically possible to achieve virtually perfect weight estimation with ideal habitus assessment. However, it is unclear whether it is possible in practice to differentiate between the relatively minor differences in external indicators of body composition that might be required to achieve this accuracy. Validated reference figural images will likely be required to enable the habitus assessment to be standardised and generalised across different populations. This technique has been validated in preliminary studies for the PAWPER XL tape, but further research is needed [30].

The ability of the PAWPER XL tape to accurately estimate FFM and IBW, in addition to being able to identify the obese children for whom this would be required for dosing calculations, was an interesting finding which might prove useful in the future. This requires further research.

## Conclusions

The association between predictive variables (age and length) and TBW, IBW, FM, and FFM showed the underlying biological limitations of using age or length alone to predict weight. None of the age-based formulas achieved a satisfactory degree of accuracy, including the age-based habitus-modified formulas of Erker. Differences between the age formulas were essentially whether the major errors were greater in lower-BMI children (APLS and Best Guess formulas) or in higher-BMI children (EPLS formula). The Broselow tape performed better than the age-formulas but it did not achieve satisfactory accuracy in estimating TBW. It was accurate in predicting IBW, however, and could be used for this purpose for drug dose calculations as long as TBW was known or estimated using another technique. The Mercy method (a dual length- and habitus-based method) demonstrated a higher accuracy in estimating TBW than the univariate methods, with fewer critical errors. Its performance was completely independent of body composition. This suggested that other patient factors or user errors might be critical determinants of its functioning accurately. It was unable to estimate IBW. The performance of the dual length- and habitus-based PAWPER XL tape was the best of all the systems. It was also the only system that had a specific mechanism to produce estimations of IBW (the HS3 weight) and FFM (the HS1 weight). The overall accuracy of estimation of TBW, IBW, and FFM were very good, even for children outside of the restrictions of other methods. Its weakest performance was in children with extreme habitus types, which was probably mostly due to errors or aberrations in the assessment of body habitus. This needs to be addressed in future research.
